# Diagnostic and Prognostic Potential of ^18^F-FET PET in the Differential Diagnosis of Glioma Recurrence and Treatment-Induced Changes After Chemoradiation Therapy

**DOI:** 10.3389/fonc.2021.721821

**Published:** 2021-10-04

**Authors:** Monica Celli, Paola Caroli, Elena Amadori, Donatella Arpa, Lorena Gurrieri, Giulia Ghigi, Patrizia Cenni, Giovanni Paganelli, Federica Matteucci

**Affiliations:** ^1^ Diagnostic Nuclear Medicine Unit, IRCCS Istituto Romagnolo per lo Studio dei Tumori (IRST) “Dino Amadori”, Meldola, Italy; ^2^ Radiology MRI Unit, IRCCS Istituto Romagnolo per lo Studio dei Tumori (IRST) “Dino Amadori”, Meldola, Italy; ^3^ Radiation Therapy Unit, IRCCS Istituto Romagnolo per lo Studio dei Tumori (IRST) “Dino Amadori”, Meldola, Italy; ^4^ Oncology Unit, IRCCS Istituto Romagnolo per lo Studio dei Tumori (IRST) “Dino Amadori”, Meldola, Italy

**Keywords:** ^18^F-FET PET, treatment-related changes, treated gliomas, metabolic tumor volume, total tumor metabolism

## Abstract

**Background:**

MRI-based differential diagnosis of glioma recurrence (GR) and treatment-induced changes (TICs) remain elusive in up to 30% of treated glioma patients. We aimed to determine ^18^F-FET PET diagnostic performance in this clinical scenario, its outcome dependency on established prognostic factors, optimal ^18^F-FET semi-quantitative thresholds, and whether ^18^F-FET parameters may instantly predict progression-free survival (PFS) and overall survival (OS).

**Methods:**

We retrospectively analyzed 45 glioma patients treated with chemoradiation therapy (32 males; mean age: 51 years, glioma grade: n=26 WHO4; n=15 WHO3; n=4 WHO2) who underwent ^18^F-FET PET to resolve differential diagnosis of GR and TICs raised by MRI performed in the preceding 2 weeks and depicting any of the following changes in their radiation field: volumetric increase of contrast-enhancing lesions; new contrast-enhancing lesion; significant increase in T2/FLAIR non-enhancing lesion without reducing corticosteroids. ^18^F-FET PET outcome relied on evaluation of maximum tumor-to-brain ratio (TBRmax), time-to-peak (TTP), and time-activity curve pattern (TAC). Metabolic tumor volume (MTV) and total tumor metabolism (TTM) were calculated for prognostic purposes. Standard of reference was repeat MRI performed 4–6 weeks after the previous MRI. Non-parametric statistics tested ^18^F-FET-based parameters for dependency on established prognostic markers. ROC curve analysis determined optimal cutoff values for ^18^F-FET semi-quantitative parameters. ^18^F-FET parameters and prognostic factors were evaluated for PFS and OS by Kaplan-Meier, univariate, and multivariate analyses.

**Results:**

^18^F-FET PET sensitivity, specificity, positive predictive value, negative predictive value were 86.2, 81.3, 89.3, 76.5%, respectively; higher diagnostic accuracy was yielded in IDH-wild-type glioma patients compared to IDH-mutant glioma patients (sensitivity: 81.8 *versus* 88.9%; specificity: 80.8 *versus* 81.8%). KPS was the only prognostic factor differing according to ^18^F-FET PET outcome (negative *versus* positive). Optimal ^18^F-FET cutoff values for GR were TBRmax ≥ 2.1, SUVmax ≥ 3.5, and TTP ≤ 29 min. PFS differed based on ^18^F-FET outcome and related metrics and according to KPS; a different OS was observed according to KPS only. On multivariate analysis, ^18^F-FET PET outcome was the only significant PFS factor; KPS and age the only significant OS factors.

**Conclusion:**

^18^F-FET PET demonstrated good diagnostic performance. ^18^F-FET PET outcome and metrics were significantly predictive only for PFS.

## 1 Introduction

Gliomas are the most common primary brain malignancies, predominantly presenting as high-grade infiltrative tumors, almost ineluctably relapsing after multimodal treatment, which combines maximal surgical safe resection, concomitant chemoradiation, and adjuvant chemotherapy (1, 2).

After treatment, detection of glioma recurrence (GR) currently relies on RANO criteria integrating magnetic resonance imaging (MRI), patient performance status, and dosage of steroids.

MRI findings defining GR include the following: (i) ≥25% increase in the products of perpendicular diameters of measurable enhancing lesions compared with their smallest measurements obtained either at baseline (if no decrease) or at best response; (ii) the emergence of any new lesions; (iii) a significant increase in T2/FLAIR non-enhancing lesions on stable or increasing doses of corticosteroids compared with baseline scan or best response after initiation of therapy. Such MRI changes, however, are not strictly tumor specific (3, 4).

A proportion of 9–30% of treated glioma patients develops treatment-induced changes (TICs) simulating GR, potentially leading to misdiagnosis and inappropriate treatment modifications. TICs mimicking GR principally include two distinct patterns: pseudo-progression (PSP) and radio-necrosis (RN). PSP reflects inflammation and transiently increased permeability of tumor vasculature occurring within the first 6 months after chemoradiation appearing in the therapy field as new or increasing contrast-enhancing lesions and decreasing over time without any therapy change. RN lesions, implying brain tissue injury, necrosis, and vascular thrombosis, generally occur in the white matter not earlier than 1 year after having received any radiation or re-irradiation and generally do not subside (5–8).

Since TICs may mimic GR on conventional MRI, the ultimate diagnosis of TICs is retrospective as it requires a repeat MRI to be performed 4 weeks later demonstrating no further significant worsening of previously noted suspicious findings. This verification approach in two times may result in delayed diagnosis of GR.

In the attempt to promptly and confidently discriminate GR and TICs the PET-RANO and EANO working groups recommended evidence-based clinical use of amino-acid PET imaging as a metabolic integration to MRI (9).

The tyrosine analog ^18^F-FET is an ^18^F-labeled PET tracer highly specific for glioma. ^18^F-FET uptake mechanism is driven by glioma overexpression of the active transmembrane L-type amino-acid transporter 2 (LAT2) and, to a minor extent, the lesser glioma-specific LAT1. An artificial amino-acid, ^18^F-FET is not further metabolized within the cell and does not serve as substrate to protein synthesis. ^18^F-FET uptake in inflammatory cells is low and negligible in the healthy brain. A large neutral amino-acid, ^18^F-FET cannot cross the intact brain-blood-barrier (BBB), and glioma ^18^F-FET uptake is not significantly influenced by changes in the BBB permeability (10–16).

These desirable features have led to wide clinical application of ^18^F-FET-based imaging in glioma patients. With regards to differentiation of GR and TICs, few published studies, mainly retrospective and on small-sized cohorts of patients, documented high diagnostic accuracy of ^18^F-FET PET imaging, encouraging its integration into the latest RANO working group guidelines and the drafting of intersocietal EANM/EANO/RANO practice guidelines and SNMMI procedure standards (17–25).

In this retrospective study, we aim to evaluate the diagnostic performance of PET-RANO-compliant ^18^F-FET semiquantitative parameters in distinguishing TICs and GR in pretreated glioma patients with MRI findings suggestive, but not conclusive, for GR and the association of ^18^F-FET outcome with established prognostic features. Additionally, we explore the potential prognostic value of ^18^F-FET-based semiquantitative parameters.

## 2 Materials and Methods

### 2.1 Ethics Statement

The present study involved the retrospective analysis of data from a study approved by the Ethics Committee IRST IRCCS AVR (approval number L2P1912 of 06/10/2015) and conducted in accordance with the Declaration of Helsinki and subsequent revisions. In accordance with the Italian legislation, written informed consent for observational retrospective studies conducted in Scientific Institutes for Research, Hospitalization and Healthcare (IRCCS) was not required.

### 2.2 Patients

For this retrospective analysis, we searched our database (time span: October 2016–December 2020) for all treated glioma patients who were referred by the Area Vasta Romagna Neuro-Oncology Multidisciplinary Team for ^18^F-FET PET imaging to assist MRI in differentiating GR and TICs.

Forty-five patients (32 males, 13 females; mean age: 51 years; range: 21–76) with treated gliomas were extracted. Of these, 26 patients had glioblastoma WHO4 (n=21 IDH-wildtype), 11 patients had astrocytoma (n=7 WHO3, of these 5 IDH-wildtype; n=4 WHO2, of these 3 IDH-wildtype), and 8 oligodendroglioma WHO3 (n=8 IDH-mutant). MGMT promoter methylation was present in 10/26 glioblastomas, in 4/11 astrocytomas, and in 2/8 oligodendrogliomas.

Out of 45 patients, 40 received primary surgery (grossly total resection in 16 patients; partial resection in 24 patients); in all patients, ^18^F-FET PET was performed at least 12 weeks after any type of radiation therapy (**Table 1**).

**Table 1:** Cohort characteristics.

Patients characteristics	Number/(proportion)
Patients	45
Gender (male/female)	(32/13)
Median age; range	55 years; 21–76 years
Median KPS; range	90; 50–100
**Glioma characteristics**	
WHO IV glioblastoma	26
IDH wild-type/IDH mutant	(21/5)
MGMT methylated/non-methylated	(10/16)
WHO grade III gliomas	15
anaplastic astrocytoma	7
IDH wild-type/IDH mutant	(5/2)
MGMT methylated/non-methylated	(1/6)
oligodendroglioma	8
IDH wild-type/IDH mutant	(0/8)
MGMT methylated/non-methylated	(2/6)
WHO grade II astrocytoma	4
IDH wild-type/IDH mutant	(3/1)
MGMT methylated/non-methylated	(1/3)
Patients who had experienced glioma relapses prior to ^18^F-FET PET	(34/45)
**Treatments**	
Patients who received primary surgery:	40
partial resection	24
grossly total resection	16
Patients who did not receive primary surgery (biopsy only)	5
Patients who received first-line chemoradiation therapy	45
Further treatments prior to ^18^F-FET PET	
surgical re-resection	6
re-irradiation	24
chemotherapy	20
temozolomide	19
PCV	3
anti-VEGF	1
Median time from last RT to ^18^F-FET PET; interquartile range	14 months; 5–22 months

IDH, isocitrate dehydrogenase; MGMT, O[6]-methylguanine-DNA methyltransferase; KPS, Karnofsky Performance Scale; PCV, procarbazine, lomustine, and vincristine; anti-VEGF, anti-vascular endothelial growth factor.

### 2.3 MR Imaging

The MRI scans performed prior to ^18^F-FET PET and afterward for clinical monitoring consisted of T1-weighted before and after contrast agent administration, T2-weighted/FLAIR, and diffusion-weighted imaging. Two neuroradiologists (CeP with 20 years’ experience and AE with 5 years’ experience) reported all post-radiation therapy MRI scans according to RANO interpretation criteria and reviewed MR images at multidisciplinary meetings. Suggestive MRI findings for GR included evidence of size-increasing contrast-enhanced lesions with at least 10 mm diameter (i.e., ≥25% increase in the product of perpendicular diameters compared with the nadir measurement obtained after radiation therapy); evidence of any new lesion within the radiation therapy field; significant increase in T2/FLAIR non-enhancing lesions compared with best response after therapy start and without decreasing corticosteroids. No significant clinical deterioration coexisted at the first evidence of the abovementioned findings.

### 2.4 ^18^F-FET PET/CT Imaging

^18^F-FET was commercially shipped with marketing authorization for the appropriate clinical indication of differentiating GR from TICs. ^18^F-FET PET scans were performed within 2 weeks from the MRI depicting dubious findings for GR. ^18^F-FET PET were acquired on a Biograph mCT Flow 64 PET/CT system (Siemens Healthineers, Germany).

Mean ^18^F-FET activity of 3MBq/Kg of body weight was intravenously administered in bolus, and simultaneously, a 40-minute three-dimensional dynamic brain PET/CT scan was started. The dynamic brain ^18^F-FET PET acquisition consisted of 35 frames (12 frames of 1 s, 6 frames of 10 s, 6 frames of 30 s, 5 frames of 1 min, and 6 frames of 5 min).

Late static brain images (summed images from 20 to 40 minutes post-injection) were generated from each dynamic ^18^F-FET PET scan.

^18^F-FET PET/CT images and the most recent MRI scan were reviewed on a syngo.via platform (Siemens Healthineers, Germany). Late static ^18^F-FET PET images were rigidly co-registered with contrast-enhanced T1-weighted MRI images for lesion correlation.

^18^F-FET PET scans were interpreted according to the joint practice guidelines collaboratively developed by the European Association of Nuclear Medicine (EANM), the Society of Nuclear Medicine and Molecular Imaging (SNMMI), the European Association of Neuro Oncology (EANO), and the working group for Response Assessment in Neuro Oncology with PET (PET-RANO).

A three-dimensional VOI with a 1.6 cm diameter was drawn on the ^18^F-FET PET hottest index lesion to measure the SUVmax. The SUVmean of contralateral healthy brain tissue, measured with a similar VOI including gray and white matter, was used to generate the maximum tumor-to-background ratio (TBRmax). The index lesion VOI was copied onto the dynamic ^18^F-FET acquisition to plot the time-activity curve (TAC), to extract the time-to-peak (TTP, minutes from the start of dynamic acquisition up to lesion maximum SUVpeak), and to draw the TAC curve.

^18^F-FET PET was considered positive for GR if the TBRmax was superior to the established cutoff value of 1.9 in the late period after radiation therapy (i.e., after 12 weeks from completion of RT). Early TTP (<20 min) and a TAC pattern with early peak and either wash-out (TAC pattern 1) or plateau (TAC pattern 2) were regarded as ancillary support of GR; late TTP (>20 min) with an accumulating curve pattern (TAC pattern 3) was deemed not suggestive of GR.

^18^F-FET PET semiquantitative parameters SUVmax, TBRmax, and TTP were evaluated by receiver-operating-characteristic curve analysis to identify the optimal threshold values and their diagnostic performance.

In ^18^F-FET PET scans documenting lesions consistent with GR, semiquantitative volumetric parameters were calculated for prognostic purposes. Metabolic tumor volume (MTV) and total tumor metabolism (i.e., TTM = MTV × lesion SUVmean) were semiautomatically calculated by drawing a VOI around the positive lesion on late static ^18^F-FET PET, encompassing all SUVmax values returning a pathological TBRmax (i.e., TBRmax > 1.9).

To do so, reference minimum pathological SUV was obtained by multiplying the healthy brain SUVmean by the pathological TBRmax cutoff value of 1.9 (i.e., reference minimum pathological SUV = healthy brain SUVmean × 1.9). The resulting SUVmax value was considered the reference minimum pathological value for MTV contour thresholding.

### 2.5 Standard of Reference

#### 2.5.1 Diagnosis of GR *versus* TIC

RANO-compliant diagnosis of GR in this study consisted in further worsening of MRI findings previously deemed suggestive for GR on the confirmatory MRI scan performed 4–6 weeks later.

The diagnosis of TICs was inferred whenever the initially worsening MRI findings stabilized on the subsequent confirmatory MRI scan. In six patients the histology results (surgical re-resection in five patients; biopsy in one patient) were made available for definitive confirmation of viable GR.

Clinical follow-up was interrogated for excluding any prior decrease or discontinuation of corticosteroids.

#### 2.5.2 PFS and OS Calculation

PFS was computed from the date of ^18^F-FET PET imaging to RANO-compliant evidence of confirmed progressive disease on MRI, namely, to the date of the confirmatory MRI documenting a second consecutive >25% increase in the product of lesion perpendicular diameters or any following MRI evidence of unequivocal GR. Follow-up MRI scans were performed every 2–4 months according to the disease status and clinical course.

Patients who did not meet RANO criteria for MRI-based GR or who were alive at last available clinical assessment were considered censored without event for PFS and OS, respectively.

### 2.6 Statistical Analysis

Statistical analysis was carried out with StatsDirect software. Diagnostic performance was calculated with 95% confidence intervals.

Shapiro-Wilk test was used to test data normality distribution. A two-sided Fisher’s Exact test was used to test whether the ^18^F-FET PET outcome (positive/negative) was significantly different according to dichotomous prognostic features, namely, IDH status (mutant *versus* wild-type) and MGMT methylation (methylated *versus* unmethylated).

A two-sided Mann-Whitney U test was used to define whether ordinal prognostic factors such as patient Karnofsky performance status (KPS), glioma WHO grade, and history of prior glioma relapses were randomly represented in ^18^F-FET PET outcome.

^18^F-FET parameters SUVmax, TBRmax, TTP, and TAC pattern of true positive glioma lesions were tested for any significant differences based on glial phenotype (Mann-Whitney) and WHO grade (Kruskal–Wallis).

Survival analysis for PFS and OS was carried out, and a log-rank test was used to identify different survival distributions based on ^18^F-FET PET outcome and its related metrics and on other available prognostic factors. Cox-regression analysis was used to test the significance of PFS and OS hazard ratios for parameters found to significantly impact survival.

Results obtained by non-parametric tests and by survival analyses considered an α-error of 0.05 and reached statistical significance at p < 0.035.

## 3 Results

Out of 45 patients presenting MRI findings suggestive for GR, 29 met RANO criteria for GR on subsequent MR evaluation performed 4–6 weeks later; of these, 25 were correctly identified by ^18^F-FET PET (n = 12 WHO grade IV, n = 11 WHO grade III, n = 2 WHO grade II). The remaining 16 patients did not show immediate progression and were cautiously considered to harbor TICs; of these 16, ^18^F-FET PET correctly identified 13 cases (n = 7 WHO grade IV, n = 4 WHO grade III, n = 2 WHO grade II).

In four cases ^18^F-FET tested falsely negative. Subsequent MRI documented further increase in size of the previously noted MRI suspicious findings. Two patients experienced recurrence of GBM (IDH wild-type), and two patients had recurrent ODG (IDH-mutant). All four patients were off systemic treatment at time of ^18^F-FET PET. One patient underwent re-resection confirming recurrence of GBM, two patients received re-irradiation of the growing lesions, and one patient was started on chemotherapy.

^18^F-FET resulted falsely positive in three patients with IDH wild-type GBM. Follow-up MRI demonstrated stability of previously noted increasing lesions.

Overall, ^18^F-FET PET diagnostic performance in differentiating GR and TICs returned sensitivity (SE) of 86.2% (95% CI: 68.3–96.1%), specificity (SP) of 81.3% (95% CI: 54.4–96.1%), positive predictive value (PPV) of 89.3% (95% CI: 71.8–97.7%), negative predictive value (NPV) of 76.5% (95% CI: 50.1–93.2%), positive likelihood ratio of 4.6 (95% CI: 2.0–13.2), and negative likelihood ratio of 0.2 (95% CI: 0.1–0.4).

^18^F-FET PET diagnostic performance for detecting GR resulted higher for IDH-wild-type gliomas than IDH-mutant gliomas (SE: 88.9 *versus* 81.8%; SP: 81.8 *versus* 80.8%; PPV: 88.9 *versus* 90%; NPV: 81.8 *versus* 66.7%).

^18^F-FET PET outcome for GR (positive/negative) was not significantly different considering the glioma IDH status (p-value: 0.894) and MGMT methylation status (p-value: 0.996).

Patients’ KPS significantly differed according to ^18^F-FET PET outcome (p-value: 0.025), whereas glioma WHO grade, history of previous relapses, and age did not.

^18^F-FET PET SUVmax, TBRmax, TTP, and TAC pattern for true positive lesions did not significantly differ across different glial phenotypes, WHO grades, nor did between IDH wild-type and mutant status and between MGMT methylated and unmethylated status (**Table 2**).

**Table 2:** Static semiquantitative ^18^F-FET PET parameters.

	SUVmax ± SD	p-value	TBRmax ± SD	p-value
True Glioma Recurrence				
Glial phenotype				
astrocytic	3.7 ± 1.7	0.591	3.4 ± 1.1	0.652
oligodendroglial	4.2 ± 1.7		3.1 ± 0.6	
WHO grade				
II	2.4 ± 1.7	0.542	3.1 ± 1.3	0.557
III	3.9 ± 1.5		3.1 ± 0.9	
IV	3.9 ± 1.9		3.6 ± 1.1	
IDH				
wild-type	3.7 ± 1.7	0.782	3.3 ± 1.1	0.857
mutant	3.9 ± 1.9		3.4 ± 0.9	
MGMT promoter				
methylated	3.7 ± 1.4	0.904	3.1 ± 1.0	0.267
unmethylated	3.8 ± 1.9		3.5 ± 1.0	
Treatment-induced changes	2.5 ± 0.5		1.9 ± 0.4	

ROC analysis returned an optimal TBRmax cutoff value of 2.1 to discriminate GR and TICs (SE: 79.3%; SP: 75%; PPV: 85.2%; NPV: 66.7%; area-under-curve AUC: 78%). The best cutoff for SUVmax was 3.5 (SE: 48.3%; SP: 93.8%; PPV: 93.3%; NPV: 50%; area-under-curve AUC: 71.1%) and for TTP at 29 min (SE: 68.8%; SP: 82.8%; PPV: 85.2%; NPV: 66.7%; area-under-curve AUC: 70.4%). Cutoff values for volumetric parameters MTV (≥0.15 ml) and TTM (≥0.33 ml) were generated returning good diagnostic accuracy (SE: 86.2%; SP: 81.3%; AUC: 85.4%) (**Table 3**).

**Table 3:** GR diagnostic performance of static and dynamic ^18^F-FET PET parameters.

	SUVmax	TBRmax	TTP	MTV	TLM
Cutoff Value	≥3.5	≥2.1	≤29 min	≥0.15 ml	≥0.33 ml
Sensitivity [95% CI]	48.3 [29.4–67.5]	79.3 [60.3–92.0]	68.8 [41.3–89.0]	86.2 [68.3–96.1]	86.2 [68.3–96.1]
Specificity [95% CI]	93.8 [69.8–99.8]	75.0 [47.6–92.7]	82.8 [64.2–94.2]	81.3 [54.3–95.9]	81.3 [54.3–95.9]
AUC ± SE	71.1 ± 0.08	78.0 ± 0.07	70.4 ± 0.09	85.4 ± 0.07	85.4 ± 0.06

**Figure 1:**
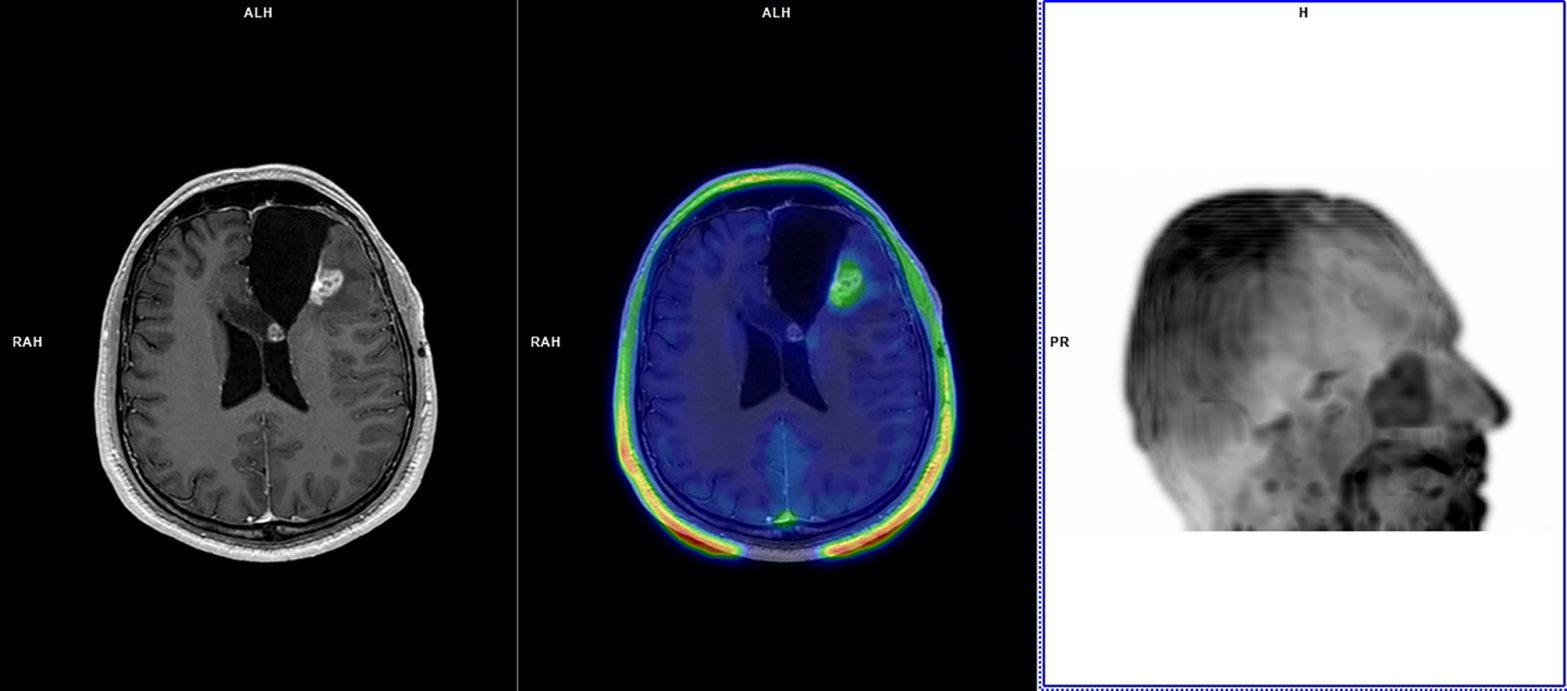
A 35-year-old patient with left frontal glioblastoma (IDH wild-type; MGMT methylated) treated with partial resection in August 2017, chemoradiation (completed in November 2017), and four cycles of adjuvant temozolomide (last cycle in March 2018). In August 2018, left frontal cavity re-irradiation was performed for glioblastoma relapse. Follow-up MRI scans in November 2018 demonstrated an increasing pseudonodular area of contrast-enhancement along the left frontal surgical cavity without functional indices abnormalities, deemed dubious for GR. ^18^F-FET PET performed in December 2018 demonstrated faint tracer uptake at late static imaging (TBRmax: 1.6) and TAC pattern 3 with TTP at 35 min. ^18^F-FET PET was considered suggestive of TICs. MRI demonstrated disease progression in August 2019 (PFS: 8 months; OS not reached), and bevacizumab was commenced. At last MRI follow-up (January 2021) MRI demonstrated stable disease.

**Figure 2:**
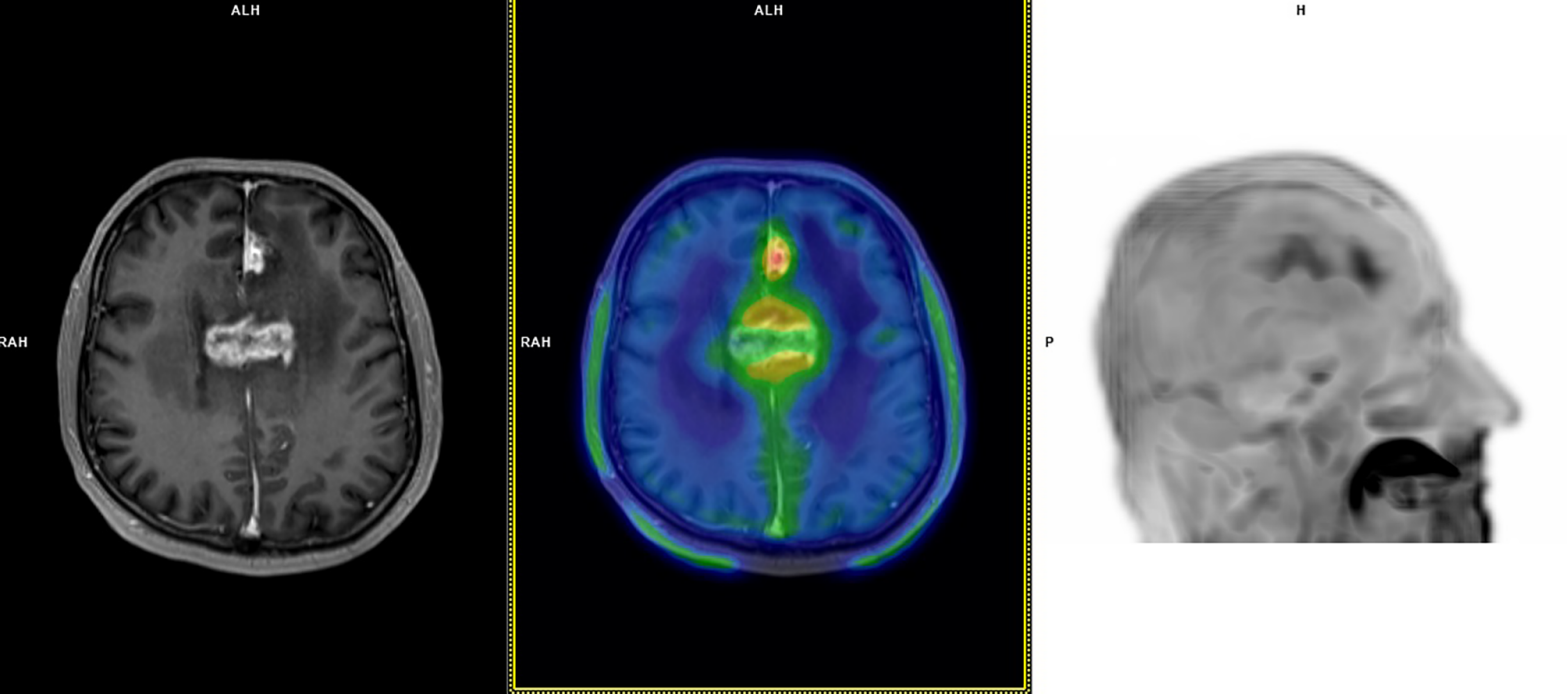
A 60-year-old patient with bioptic diagnosis of astrocytoma WHO grade 3 with lesions in the left frontal lobe and corpus callosum (IDH wild-type; MGMT methylated) treated with chemoradiation (completed in March 2018) and re-irradiation in July 2018. Follow-up MRI scans in October 2018 documented an increasing T2/FLAIR signal and irregular contrast-enhancement at the medial aspect of the left frontal lobe and corpus callosum; no diffusion restriction was seen, and perfusion weighted imaging demonstrated slight increase in Ktrans e Ve values without abnormal Vp. MRI appearances were deemed dubious for GR, favouring the hypothesis of TICs. ^18^F-FET PET performed in November 2018 demonstrated focal tracer uptake at late static imaging (TBRmax: 3.4), a TAC pattern with a TTP at 20 min and subsequent plateau (TAC pattern 2). ^18^F-FET PET was considered suggestive of GR. MRI performed in January 2019 documented GR, and chemotherapy was started. This patient died in July 2019 (PFS: 1 month; OS: 8 months).

Median follow-up time after ^18^F-FET PET was 7.5 months (interquartile range: 16.1 months). During follow-up, 31 patients eventually progressed (68.9%) and 11 patients died (24.4%). Survival univariate analysis documented statistically significant differences in PFS between ^18^F-FET positive and ^18^F-FET negative patients (PFS of 1.4 months and 14.7 months, respectively; log-rank test, p < 0.0001) and ^18^F-FET-related metrics, and between patients with KPS < 80 and KPS ≥ 80 (PFS of 1.2 months *versus* 3.8 months, respectively; log-rank test, p: 0.0002). No significant differences in PFS were observed based on the IDH status (log-rank test, p: 0.588), MGMT status (log-rank test, p: 0.263), and WHO grade III/IV *versus* grade II (log-rank test, p: 0.872) (**Table 4**) (**Figures 1** and **2**).

**Table 4:** Univariate analysis.

Parameters	Criterion	PFS		OS	
		p value	Median PFS time	p value	Median OS time
Age	≥50 years *vs.* <50 years	0.707	2.3 *vs.* 2.6 months	0.150	26.0 months *vs.* OS not reached
Resection	biopsy *vs.* complete/partial resection	0.782	2.3 *vs.* 5.5 months	0.607	26.0 months *vs.* OS not reached
WHO grade	IV *vs.* III/II	0.872	2.3 *vs.* 2.6 months	0.855	26.0 months *vs.* OS not reached
IDH status	mutant *vs.* wildtype	0.588	2.3 *vs.* 2.9 months	0.162	26 *vs.* 21.6 months
MGMT promoter	non-methylated *vs.* methylated	0.263	3.0 *vs.* 2.2 months	0.684	21.6 *vs.* 26 months
KPS	≥80 *vs.* <80	***0.0002***	3.8 *vs.* 1.2 months	***0.0013***	7.7 months *vs.* OS not reached
Prior relapses	yes/no	0.315	2.4 *vs.* 7.5 months	0.329	21.6 months *vs.* OS not reached
^18^F-FET outcome	positive *vs.* negative	***<0.0001***	1.4 *vs.* 14.7 months	0.405	16.9 *vs.* 26.1 months
SUVmax	≥3.5 *vs.* <3.5	***0.0009***	1.2 *vs.* 3.6 months	0.094	10.4 months *vs.* OS not reached
TBRmax	≥2.1 *vs.* <2.1	***<0.0001***	1.7 *vs.* 14.3 months	0.300	21.6 months *vs.* OS not reached
TTP	<29 min *vs.* ≥29 min	***0.0044***	1.4 *vs.* 10.4 months	0.307	21.6 months *vs.* OS not reached
MTV	≥0.15 ml *vs.* <0.15 ml	***0.0001***	1.4 *vs.* 14.7 months	0.365	26.1 months *vs.* 16.9 months
TTM	≥0.33 ml *vs.* <0.33 ml	***0.0001***	1.4 *vs.* 14.7 months	0.405	26.1 months *vs.* 16.9 months

IDH, isocitrate dehydrogenase; MGMT, O[6]-methylguanine-DNA methyltransferase; KPS, Karnofsky Performance

Scale; MTV, metabolic tumor volume; TTM, total tumor metabolism; PFS, progression-free survival; OS, overall survival. The bold italicized values means statistically significant.

In the sub-cohort of patients with high-performance status (i.e., KPS ≥ 80; n = 32 patients), ^18^F-FET PET outcome significantly impacted on PFS (log-rank p: 0.0005) with a median PFS of 2.4 months for patients with positive ^18^F-FET PET (n = 15) and a median PFS of 14.7 months for patients with negative ^18^F-FET PET (n = 17).

Cox-regression analysis documented statistically significant PFS positive hazard ratio for ^18^F-FET positive outcome (HR: 8.91; p = 0.0005), whereas no statistical significance was found for any pathology, treatment, and patient-related features. ^18^F-FET cutoffs for volumetric parameters MTV and TTM did not significantly weigh on the PFS (HR: 1). OS-based survival analysis documented statistically significant differences only according to KPS < 80 and KPS ≥ 80 (median OS of 7.7 months and OS not reached; log-rank test, p = 0.0001). Neither ^18^F-FET PET parameters nor pathology-related features were found to impact OS. Statistically significant OS positive hazard ratios were only detected for KPS (HR: 55.98; p = 0.003) and age at the time of ^18^F-FET PET (HR: 11.4; p = 0.015) (**Table 5**).

**Table 5:** Multivariate analysis.

Parameters	Criteria	PFS [95% CI]		OS [95% CI]	
		Hazard ratio	p-value	Hazard ratio	p-value
**Age**	≥50 years	1.25 [0.54–2.89]	0.598	***11.43 [1.59–82.16]***	***0.015***
**Primary Surgery**	non-total resection	0.75 [0.15–3.73]	0.724	39.993	0.994
**WHO grade**	IV grade	0.92 [0.42–2.02]	0.834	0.40 [0.08–2.06]	0.275
**IDH status**	wild-type	0.35 [0.11–1.08]	0.069	0.07 [0.01–0.70]	0.023
**MGMT promoter**	unmethylated	1.29 [0.57–2.91]	0.543	0.47 [0.09–2.44]	0.373
**Prior relapses**	yes	0.81 [0.31–2.07]	0.656	4.20 [0.55–32.04]	0.166
**KPS**	<80	2.20 [0.87–5.53]	0.094	***55.98 [3.92–798.75]***	***0.003***
**^18^F-FET outcome**	positive	***8.91 [2.62–30.29]***	***0.0005***	0.47 [0.04–5.24]	0.539
**MTV**	≥0.15 ml	1.0 [1.0–1.0]	<0.0001	1.0 [1.0–1.0]	<0.0001
**TTM**	≥0.33 ml	1.0 [1.0–1.0]	<0.0001	1.0 [1.0–1.0]	<0.0001

IDH, isocitrate dehydrogenase; MGMT, O[6]-methylguanine-DNA methyltransferase; KPS, Karnofsky Performance Scale; MTV, metabolic tumor volume; TTM, total tumor metabolism; PFS, progression-free survival; OS, overall survival. The bold italicized values means statistically significant.

## 4 Discussion

The present study, stemming from our institution’s initial experience on a small-sized cohort of patients undergoing ^18^F-FET PET, documents a diagnostic performance, slightly inferior to evidence from previously published literature with a sensitivity of 86.2% (95% CI: 68.3–96.1%) and a specificity of 81.3% (95% CI: 54.4–96.1%).

A recent meta-analysis by de Zwart et al. analyzed 10 studies on ^18^F-FET PET (of these, three abstracts; 207 scans overall) in treated glioma patients with suspect GR, documenting a pooled sensitivity and specificity of 90% (95% CI, 81–95%) and 85% (95% CI, 71–93%). A meta-analysis from Kim et al. considering six studies on ^18^F-FET PET (212 scans overall) in this clinical scenario found a pooled sensitivity and specificity of 89% (95% CI, 82–94%) and 88% (95% CI, 76–94%) (26, 27).

A recent retrospective study on a large cohort of treated WHO II-IV glioma patients (n = 127) by Maurer et al. also documented slightly inferior accuracy, with sensitivity, specificity, and accuracy of 86, 67, and 81%, respectively. The Authors found that ^18^F-FET PET diagnostic performance, both in terms of sensitivity and specificity, was negatively affected by the presence of IDH mutations (diagnostic accuracy was 91% in IDH-wild-type gliomas and 67% in IDH-mutant gliomas; p < 0.001) (28).

In our study cohort, 16 patients (35.6% cases) had IDH-mutant gliomas, a proportion comparable to the 40% of IDH-mutant patients in Maurer’s cohort. We could observe reduced ^18^F-FET PET diagnostic accuracy for the identification of recurrent IDH-mutant gliomas compared to IDH-wild-type gliomas mainly due to decreased sensitivity (SE: 81.8 *versus* 88.9%; SP: 80.8 *versus* 81.8%). Irrespective of the IDH status, ^18^F-FET PET false negativity may depend on low cellularity of GR, on small-sized viable glioma components, on GR low or defective expression of amino-acid transporter LAT2. We speculate that the relatively higher incidence of low ^18^F-FET uptake in IDH-mutant gliomas may be consequent on metabolic reprogramming leading to reduced amino acid exchange *via* the LAT2 antiport. Glioma IDH-mutant genotype has also been found to induce immune quiescence in the tumor microenvironment, which also may account for furtherly reduced ^18^F-FET non-specific uptake (29).

All three cases of ^18^F-FET PET false positivity were IDH wild-type glioblastomas, and in one case hypofractionated re-irradiation had been previously performed. Higher rates of radionecrosis have been associated with higher radiation doses, with volume of the target lesion, and with re-irradiation (30–32).

Radiation-induced injury involves a degree of inflammatory response, ischemia, and infarction of brain areas and may result in high ^18^F-FET unspecific uptake (33–41).

As opposed to evidence from ^18^F-FET PET studies conducted on newly diagnosed gliomas, in the post-treatment scenario, we did not observe any significant dependence of ^18^F-FET metrics on WHO grade, glial type, and IDH status; this lack of association possibly depends on a more heterogeneous metabolic composition of post-treatment changes and GR.

^18^F-FET outcome (positive or negative) was significantly different between patients with KPS ≥ 80 and KPS < 80, possibly representing a degree of association with GR-related clinical deterioration.

The ROC curve analysis obtained by comparison with the standard of reference in this late post-treatment scenario (i.e., more than 12 weeks from the last radiation treatment) returned a ^18^F-FET TBRmax cutoff (≥2.1) slightly higher than that of 1.9 suggested by intersocietal practice guidelines, but equal or very similar to that found in the studies by Popperl (TBRmax ≥ 2.2), Galldiks (TBRmax ≥ 2.0), Kebir (TBRmax ≥ 2.1), Sogani (TBRmax ≥ 2.09), Bashir (TBRmax ≥ 2.0), and Maurer (TBRmax ≥ 1.95). TBRmax was found to be the most accurate ^18^F-FET semiquantitative imaging biomarker for GR compared to lesion SUVmax and TTP, returning a sensitivity, specificity, and AUC of 79.3, 75.0, 78.0%, respectively (17, 20, 22, 24, 28).

Cutoffs for the ^18^F-FET TBRmax-dependent volumetric parameters MTV and TTM were also found accurate with identical sensitivity, specificity, and AUC of 86.2, 81.3, 85.4%, respectively.

Given the possible co-existence of TICs and GR in the same patient, we introduced a new reproducible and patient-adapted method for segmenting ^18^F-FET-based MTV and compute its derived parameter TTM. First, MTV was computable only in those cases where pathological TBRmax for GR was yielded, this prerequisite increasing specificity for GR segmentation. Secondly, to draw the presumed GR-specific MTV, we exclusively considered the lesion metabolic volume encompassing all SUVmax values that divided by the healthy brain SUVmean would return a TBRmax suggestive for GR. This condition was added in order to discard SUVmax values attributable to TICs. Survival-wise, this method easily produced volumetric metabolic parameters found to negatively impact PFS but not OS.

We believe that this inability of MTV and TTM to predict OS mainly depends on the heterogeneity of ^18^F-FET PET timing in patients’ clinical histories. In their retrospective study, Bashir et al. investigated the prognostic value of ^18^F-FET-derived biological tumor volume (BTV) performed 6 months after radiation therapy completion in 146 glioblastoma patients (24). In their homogeneous cohort of patients, BTV (cutoff value = 0.55cc) was found to be a strong predictor of OS both at univariate (HR 1.303, p < 0.0001) and multivariate analyses (HR 1.339, p < 0.0001). A significant association with OS was demonstrated for patients’ performance status, whereas the extent of surgery, age, MGMT promoter status, and IDH status did not predict patients’ OS in glioma recurrence.

We caution on several limitations to this study. The retrospective design of the analysis may introduce selection bias. Only patients referred to multidisciplinary team discussions by internal medical and radiation oncologists had the chance to access FET PET imaging and exclusively in those cases where the review of MRI findings by the neuro-radiologists could not confidently discern the tumor or treatment-related nature of such findings. This preselection bias might limit the extrapolation of the present study results to the external clinical settings. In this study, the proportion of patients with confirmation of TIC was 35.5% (16 out of 45 patients), a datum falling at the highest end of the predicted incidence of pseudo-progression/radionecrosis. The standard of reference, prevalently based on repeat MRI and clinical follow-up, possibly leads to misclassification in some cases. The small sample size and its heterogeneity, the different type and number of treatments undergone by included patients. PFS and OS were calculated from the date of ^18^F-FET PET. This computation may have potentially biased the prognostic weight of well-established prognostic metrics such as age at time of ^18^F-FET (as this does not take into account the differing number of previous treatments), WHO grade, IDH status, MGMT status, and histotypes (astrocytic versus oligodendroglial).

In the present study, we could not evaluate associations between ^18^F-FET PET and advanced MRI techniques or MRI spectroscopy. A small number of retrospective studies evaluated the feasibility and clinical value of simultaneous and sequential ^8^F-FET PET/advanced MRI in differentiating GR from TIC. In a retrospective cohort of 104 treated glioma patients with suspected GR, Steidl et al. investigated the diagnostic performance of sequential perfusion-weighted MRI (PWI) and ^18^F-FET PET. The authors found that high hyperperfusion defined by normalized maximum relative cerebral blood volume (rCBVmax: 2.85) could correctly identify GR in 42.3% of cases, whereas in the remaining 57.7% of cases, its poor negative predictive value (NPV = 36%) could be surpassed by the addition of static and dynamic ^18^F-FET PET parameters, increasing diagnostic accuracy to 78%. The authors found that in the subgroup of IDH-mutant GR, PWI held superior diagnostic accuracy than ^18^F-FET PET (PWI AUC: 80%; ^18^F-FET AUC: 62%) (42).

In their retrospective study on 32 treated glioma patients undergoing simultaneous ^18^F-FET PET/MRI and MRI spectroscopy to identify suspect GR, Sogani et al. found that the addition of ^18^F-FET parameters slightly improved sensitivity of advanced MRI and magnetic resonance spectroscopy (MRS), with sensitivity, specificity, and diagnostic accuracy of 100, 85.7, and 96.87% for ^18^F-FET PET/MRI and 96, 85.7, and 93.75% for advanced MRI and MRS (43).

The authors found significant positive correlations between TBR_max_ and TBR_mean_ and normalized rCBV_mean_ and significant negative correlations between between TBR_max and_ mean apparent diffusion coefficient ADC_mean_. The spectroscopy-derived Choline/Creatinine ratio alone showed a sensitivity, specificity, and accuracy of 96, 85.7, 93.75%, respectively. No significant correlation was found between ^18^F-FET TBRmax and TBR_mean_ and Choline/Creatinine ratio.

Despite the encouraging results of multimodal ^18^F-FET PET/MRI, there remains a highly variable range of PWI parameter cutoffs, which strongly limits semiquantitative PWI reproducibility across centers.

Prospectively designed studies in larger and more homogeneous cohorts of pretreated glioma patients, possibly conjugating ^18^F-FET PET and advanced MRI and MRS biomarkers, would better elucidate the complementary interplay of metabolic abnormalities underlying glioma recurrence and its prognosis.

## 5 Conclusions

^18^F-FET PET demonstrated good diagnostic performance in discriminating GR and TICs, yielding higher accuracy in the assessment of IDH-wildtype gliomas as compared to IDH-mutant gliomas.

Among all considered prognostic parameters, only KPS was found to significantly differ according to ^18^F-FET PET outcome.

^18^F-FET PET outcome significantly predicted PFS in the whole studied cohort as well as in the sub-cohort of patients with high KPS.

Despite the heterogeneity of previous treatments, histological and molecular types of gliomas, and patient performance status, ^18^F-FET PET-positive outcome remained the only predictor of PFS at multivariate analysis.

Neither ^18^F-FET PET outcome/metrics nor volumetric metabolic parameters MTV and TTM were found instantly predictive of OS. KPS and age were the only predictors of OS in this cohort of patients.

## Data Availability Statement

The raw data supporting the conclusions of this article will be made available by the authors, without undue reservation.

## Ethics Statement

The studies involving human participants were reviewed and approved by IRST IRCCS AVR. Written informed consent for participation was not required for this study in accordance with the national legislation and the institutional requirements.

## Author Contributions 

MC, PCa, and FM reported ^18^F-FET PET scans, designed the research, analyzed the results, and wrote the manuscript. EA and PCe reported brain MRI scans, verified the analytical methods, and supervised the findings of this work. DA, LG, GG, and GP contributed to the design and clinical implementation of this research. All authors discussed the results and contributed to the draft of this manuscript. All authors contributed to the article and approved the submitted version.

## Conflict of Interest

The authors declare that the research was conducted in the absence of any commercial or financial relationships that could be construed as a potential conflict of interest.

## Publisher’s Note

All claims expressed in this article are solely those of the authors and do not necessarily represent those of their affiliated organizations, or those of the publisher, the editors and the reviewers. Any product that may be evaluated in this article, or claim that may be made by its manufacturer, is not guaranteed or endorsed by the publisher.
